# Identification of dysregulated lncRNAs profiling and metastasis‐associated lncRNAs in colorectal cancer by genome‐wide analysis

**DOI:** 10.1002/cam4.1168

**Published:** 2017-08-31

**Authors:** Yan Chen, Xiang Yu, Yongcan Xu, Hua Shen

**Affiliations:** ^1^ Department of General Surgery Huzhou Central Hospital Huzhou China; ^2^ Department of General Surgery The Affiliated Yantai Yuhuangding Hospital of Qingdao University Yantai China

**Keywords:** Biomarker, clinical relevance, CRC, lncRNAs, metastasis

## Abstract

The colorectal cancer (CRC) is one of the leading causes of cancer‐related death worldwide, but the pathogenesis of CRC remains not well‐known. Increasing studies have highlighted the critical roles of long noncoding RNAs (lncRNAs) in tumorigenesis and cancer cells metastasis, however, the expression pattern, biological roles of lncRNAs, and the mechanisms responsible for their function in CRC remain elusive. In this study, we performed a genome‐wide comprehensive analysis of lncRNAs profiling and clinical relevance to identify novel lncRNAs for the further study in CRC. RNA sequencing and microarray data obtained from The Cancer Genome Atlas (TCGA) and Gene Expression Omnibus (GEO) were annotated and analyzed to find differentially expressed lncRNAs in CRC. Analysis of these datasets revealed that hundreds of lncRNAs expression are dysregulated in CRC tissues when compared with normal tissues. By genomic variation analyses, we identified that some of these lncRNAs dysregulation is associated with the copy number amplification or deletion. Moreover, many lncRNAs expression levels are significantly associated with CRC patients overall and recurrence‐free survivals, such as H19, LEF1‐AS1, and RP11‐296E3.2. Furthermore, we identified one liver metastasis‐associated lncRNA termed LUCAT1 in CRC by analyzing lncRNAs expression profiles in the CRC tissues from patients with liver metastasis compared with the CRC tissues without metastasis. Finally, loss‐of‐function assays determined that knockdown of LUCAT1 could impair CRC cells invasion. Taken together, aberrantly expressed lncRNAs may play critical roles in the development and liver metastasis of CRC, and our findings may provide useful resource for identification of novel biomarkers of CRC.

## Introduction

Colorectal cancer (CRC) is one of the most common malignancies worldwide, and remains the fourth most common malignant cancer in China, with a progressively increasing incidence 1, 2, 3. Although about 90% of CRC patients with early stage can be cured due to the recent advances in chemotherapy and molecular target treatment, the survival of rest of the CRC patients is closely related to the metastasis, especially to the liver 4, 5. In spite of recent studies that have revealed the alterations in protein coding genes which functions as oncogene or tumor suppressor in CRC, the molecular and genetic basis of CRC remain unclear. Therefore, better understanding the molecular mechanisms that control CRC cells growth and metastasis is imperative for developing early diagnostic strategies as well as individualized therapy.

Over the past decades, the fast improvement of next‐generation sequencing technique and advances in the bioinformatics methods have led to the achievement of human whole genome sequencing and ENCODE (Encyclopedia of DNA Elements) Consortium, which has revealed that only less than 2% of human genome are protein coding genes, while the majority yields thousands of noncoding transcripts 6, 7, 8. These transcripts are processed into noncoding RNAs, such as microRNA, pseudogene, and long noncoding RNAs (lncRNAs). Recently, increasing evidence have demonstrated that lncRNAs participate in many cellular process, such as X chromosome imprinting, muscle cells differentiation, immune response, cell fate decision, cancer cells proliferation, metastasis and drug resistance 9, 10, 11. Moreover, large‐scale RNA sequencing in multiple cancer tissues and normal tissues revealed that lots of lncRNAs are differently expressed in tumor tissues compared with normal tissues, which indicates that these dysregulated lncRNAs might be involved in tumorigenesis and cancer progression 12. Importantly, some lncRNAs were found to function as oncogenes or tumor suppressors through interacting with RNA‐binding proteins and epigenetically repressing or activating underlying targets transcription, or acting as miRNAs “sponge” 13. For example, Sun et al. reported that gastric cancer‐associated lncRNA HOXA11‐AS promotes cell proliferation and invasion by scaffolding the PRC2, LSD1, and DNMT1, and functioning as competing endogenous RNA for miR‐1297 in gastric cancer 14.

In case of CRC, several lncRNAs functions and their mechanisms response for the regulation of underlying targets have been uncovered. For example, overexpressed lncRNA UCC promotes CRC progression by acting as an endogenous sponge via competing for miR‐143 15; lncRNA CASC11 could interact with hnRNP‐K, and thereby activate the WNT/*β*‐catenin pathway to promote CRC cells growth and metastasis 16. In addition, downregulated lncRNA RP11‐708H21.4 is related with CRC patients poor prognosis and promotes tumorigenesis through regulation of AKT/mTOR pathway 17, and lncRNA CRNDE promotes CRC cell proliferation and chemoresistance through miR‐181a‐5p‐mediated regulation of Wnt/*β*‐catenin signaling 18. Although some lncRNAs biological function and mechanisms have been characterized in CRC, the expression pattern and clinical relevance of most lncRNAs in CRC remain unknown. To uncover the lncRNAs profiling and identify CRC liver metastasis‐associated lncRNAs, we performed genome‐wide analyses to determine lncRNAs profiles in CRC by analyzing TCGA RNA sequencing data in CRC tissues and adjacent normal samples, and three gene profiling datasets from GEO. The present study reveals the aberrant lncRNAs expression in CRC, which may provide useful candidates for CRC diagnosis and treatment.

## Materials and Methods

### TCGA and public gene expression datasets analyses

The TCGA CRC tumor tissues and paired normal tissue samples RNA sequencing data and corresponding clinical data were download from http://ibl.mdanderson.org/tanric/_design/basic/download.html. Another four public CRC RNA sequencing and microarray datasets (GSE50760 19, GSE70880 20, GSE41657, and GSE95423) were downloaded from Gene Expression Omnibus (GEO). LncRNAs expression profiling of GEO microarray datasets was analyzed using the Agilent‐014850 Whole Human Genome Microarray 4x44K G4112F, Agilent‐045997 Arraystar human lncRNA microarray V3, and Agilent‐038314 CBC *Homo sapiens* lncRNA + mRNA microarray V2.0. These microarray data were preprocessed by using R software and packages.

### Genomic variation analysis

The raw CRC tissues somatic gene copy number variation data were downloaded from Broad GDAC FireBrowser website. Then, the significantly recurrent lncRNAs genomic regions copy number amplifications and deletions were determined by using GISTIC 2.0. The amplification and deletion peaks with *q*s < 0.25 were considered as significant. The lncRNAs genomic regions were mapped by the GISTIC peaks. The focal/broad frequencies, number of lncRNAs in peaks, and peak *q* values were summarized at gene level.

### CRC survival associated lncRNAs analysis

To determine the relationship between lncRNAs expression levels and CRC patients overall survival (OS) and recurrence‐free survival (RFS) time and identify survival‐associated lncRNAs, the univariable Cox regression analysis was performed. Then, multivariable Cox regression analysis was conducted to evaluate each of these lncRNAs as dependent variable factor. Next, the risk score of each lncRNAs was computed, and CRC patients were divided into high‐ and low‐risk groups according to the median risk score. The lncRNAs with log‐rank *P* < 0.05 between high‐risk and low‐risk groups were considered statistically significant. R software and Bio‐conductor were used for all these analyses. *P *<* *0.05 was considered as significant.

### Cell culture and siRNA transfection

CRC cell lines SW620 and SW480 were purchased from the Type Culture Collection of the Chinese Academy of Sciences (Shanghai, China). SW620 and SW480 cells were cultured in Dulbecco's modified Eagle's medium (Invitrogen, Carlsbad, CA) supplied with 100 U/mL penicillin and 1 *μ*g/mL streptomycin (Invitrogen), 10% fetal bovine serum (Invitrogen, shanghai, China) at 37°C with 5% CO_2_. The LUCAT1 and negative control siRNAs (Invitrogen, Carlsbad, CA) were transfected into SW620 and SW480 cells using RNAiMAX (Invitrogen) according to the manufacturer's instructions. Forty‐eight hours after transfection, the cells were harvested for RNA extraction. The LUCAT1 siRNA sequences are as follows: siRNA 1#, 5′‐CCCAUCAGAAGAUGUCAGAAGAUAA‐3′; siRNA 2#, 5′‐CAAGCUCUUGCAGUCAACAAGAACU‐3′.

### RNA extraction and qRT‐PCR

SW620 and SW480 cells RNA were extracted using RNeasy Purification Kit (Qiagen), according to the manufacturer's protocol. Then, 1 *μ*g of total RNA was reverse transcribed into cDNA using PrimeScript RT Reagent Kit (TaKaRa, Dalian, China). SYBR Premix Ex Taq (TaKaRa) was used to examine LUCAT1 expression levels, and GAPDH was used as an internal control. The primer sequence of LUCAT1 is, forward 5′‐ACCAGCTGTCCCTCAGTGTTCT‐3′, reverse 5′‐AGGCCTTTATCCTCGGGTTGCCT‐3′. The primer sequence of GAPDH is, forward 5′‐AGAAGGCTGGGGCTCATTTG‐3′, reverse 5′‐AGGGGCCATCCACAGTCTTC‐3′. qRT‐PCR assays were performed on ABI7500, and comparative cycle threshold (CT) (2^−ΔΔCT^) method was conducted to analyze the data.

### Transwell assays

Transwell assays (Corning, Tewksbury, MA, 8.0‐*μ*m pores) were used to determine SW620 and SW480 cell invasive ability after transfection with LUCAT1 or negative control siRNAs. Cells (5 × 10^4^) in 350 *μ*L medium with 1% FBS were placed into the upper chamber of an insert coated with Matrigel (Sigma‐Aldrich). To the lower chamber, 700 *μ*L medium containing 10% FBS was added. After 24 h incubation, the SW620 and SW480 cells invaded through the membrane were fixed with methanol, then stained with 0.1% crystal violet, and imaged using an IX71 inverted microscope (Olympus, Tokyo, Japan).

### Statistical analysis

The Student's *t* test (two‐tailed) and one‐way ANOVA were used to analyze qRT‐PCR, and in vitro assays data using R software and Bio‐conductor. *P* < 0.05 was considered statistically significant.

## Results

### Identification of lncRNAs alterations in CRC tissues

**Figure 1:**
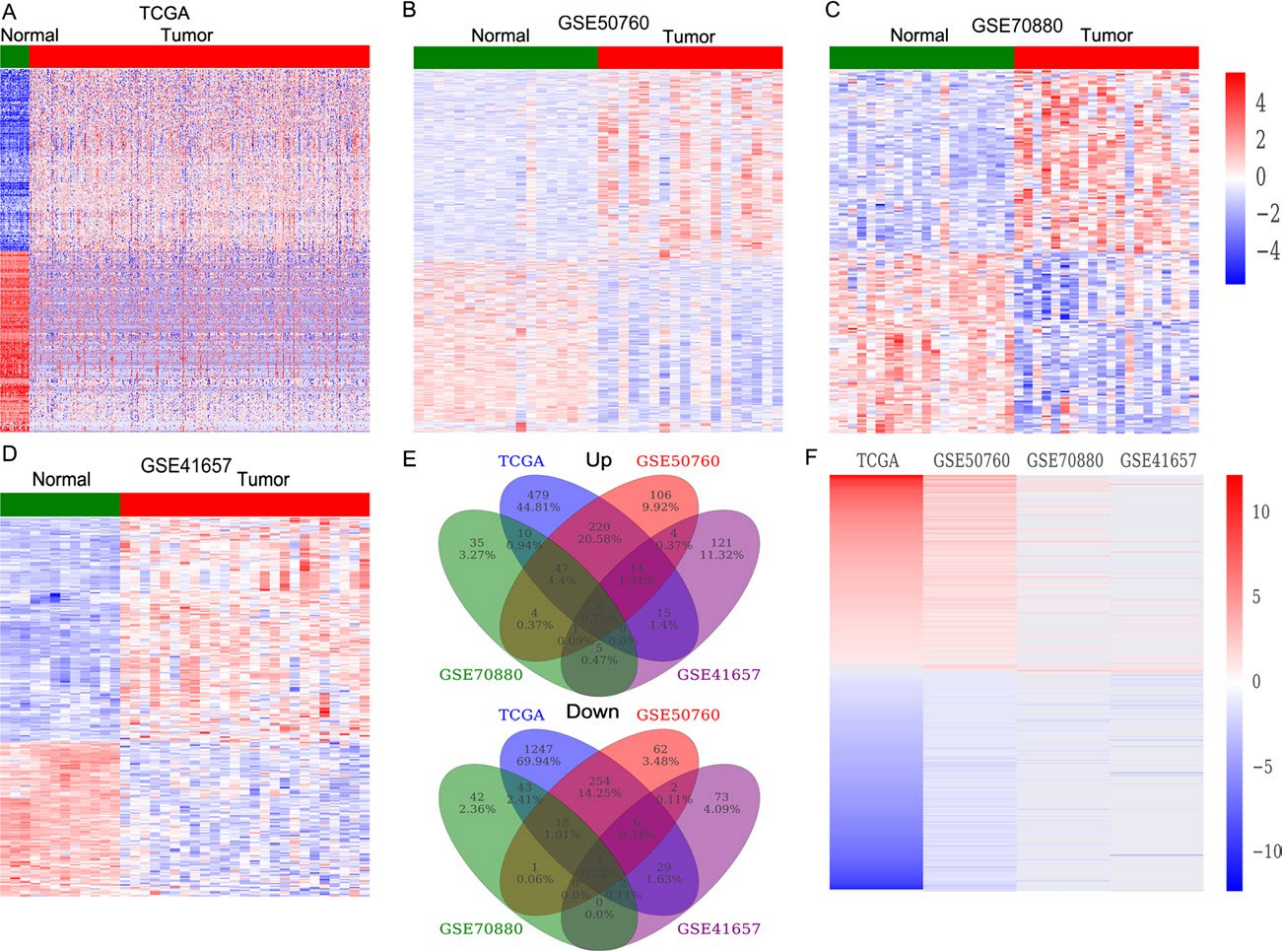
The lncRNAs profiling in CRC tissues and normal tissues. (A) Heatmap of the dysregulated lncRNAs expression in CRC and normal tissue samples was analyzed using the TCGA datasets. (B–D) Heatmap of the differentially expressed lncRNAs in CRC was analyzed using the GSE50760, GSE41657, and GSE70880 datasets. (E) Heatmap of the altered lncRNAs profiling (consistently altered at least two datasets, fold change) in TCGA, GSE50760, GSE41657, and GSE70880 datasets. (F) Venn diagram of altered lncRNAs in TCGA, GSE50760, GSE41657, and GSE70880 datasets.

To determine the lncRNA expression profiling in CRC tissues, we used the CRC and nontumor tissue samples RNA sequencing data and microarray profiling datasets (GSE50760, GSE70880, GSE41657) from TCGA and GEO. The TCGA dataset consists of 480 CRC samples and 41 normal tissue samples, while the GSE41657 dataset consists of 18 paired samples; GSE70880 consists of 20 paired samples; GSE70880 consists of 12 normal mucosae samples and 25 adenocarcinomas samples. Analysis of these datasets showed that 2396 lncRNAs expression was dysregulated in the TCGA dataset (793 upregulated and 1603 downregulated); 752 lncRNAs were differentially expressed in the GSE50760 dataset (404 upregulated and 348 downregulated); 220 lncRNAs were dysregulated in the GSE70880 dataset (110 upregulated and 110 downregulated); and 284 lncRNAs were differentially expressed in the GSE41657 dataset (168 upregulated and 116 downregulated) (Fig. 1A–D, and Table S1). Further analyses revealed that 688 lncRNAs were consistently upregulated or downregulated in at least two datasets (Fig. 1E–F, Table S2). These findings suggest that lots of lncRNAs are differentially expressed in CRC, and some of these lncRNAs may be used as novel biomarkers for CRC diagnosis.

**Figure 2:**
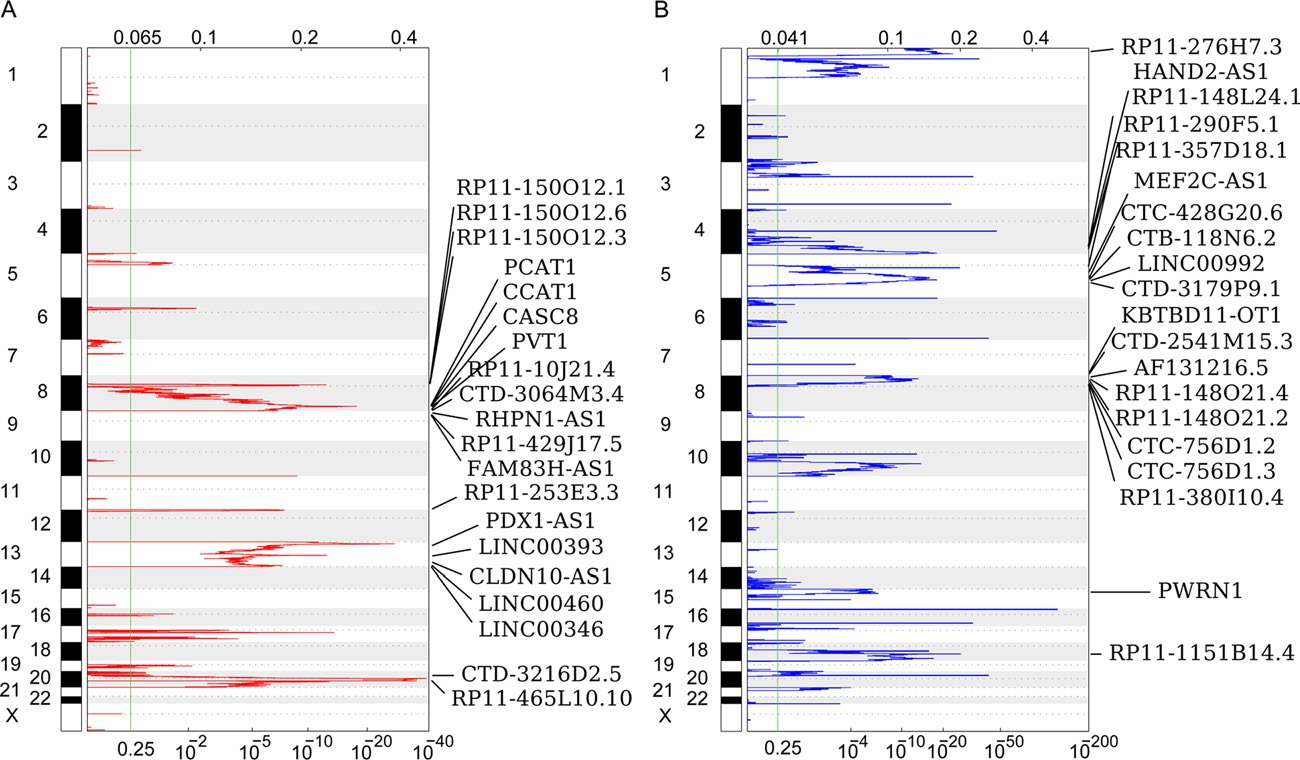
Heatmap of copy number gain and loss for lncRNAs in CRC. (A) Frequency of lncRNAs copy number gain (red) in CRC tissues and the rows are arranged according to the lncRNAs genomic locations, each of which represents an lncRNA locus. (B) Frequency of lncRNAs copy number loss (blue) in CRC tissues and the rows are arranged according to the lncRNAs genomic locations, each of which represents an lncRNA locus.

### LncRNAs somatic copy number alterations in CRC

Recently, there is evidence that indicates genomic alterations and epigenetic modifications are involved in lncRNAs disorder expression in cancer cells. Here, we analyzed the somatic copy number alterations of those differentially expressed lncRNAs in CRC using TCGA data to evaluate whether genomic alterations contribute to lncRNAs dysregulation in CRC. First, each lncRNAs gene‐containing loci SCNAs frequencies were calculated. Then, the alterations that occur in all CRC samples with *q*s < 0.25 were considered as significant alteration. The analyses results showed that there are 214 lncRNAs with frequency gain and 311 lncRNAs with frequency loss in CRC (Fig. 2A and 2B, and Table S3). These findings indicate that somatic copy number variations might involve in some lncRNAs dysregulation in CRC.

**Figure 3:**
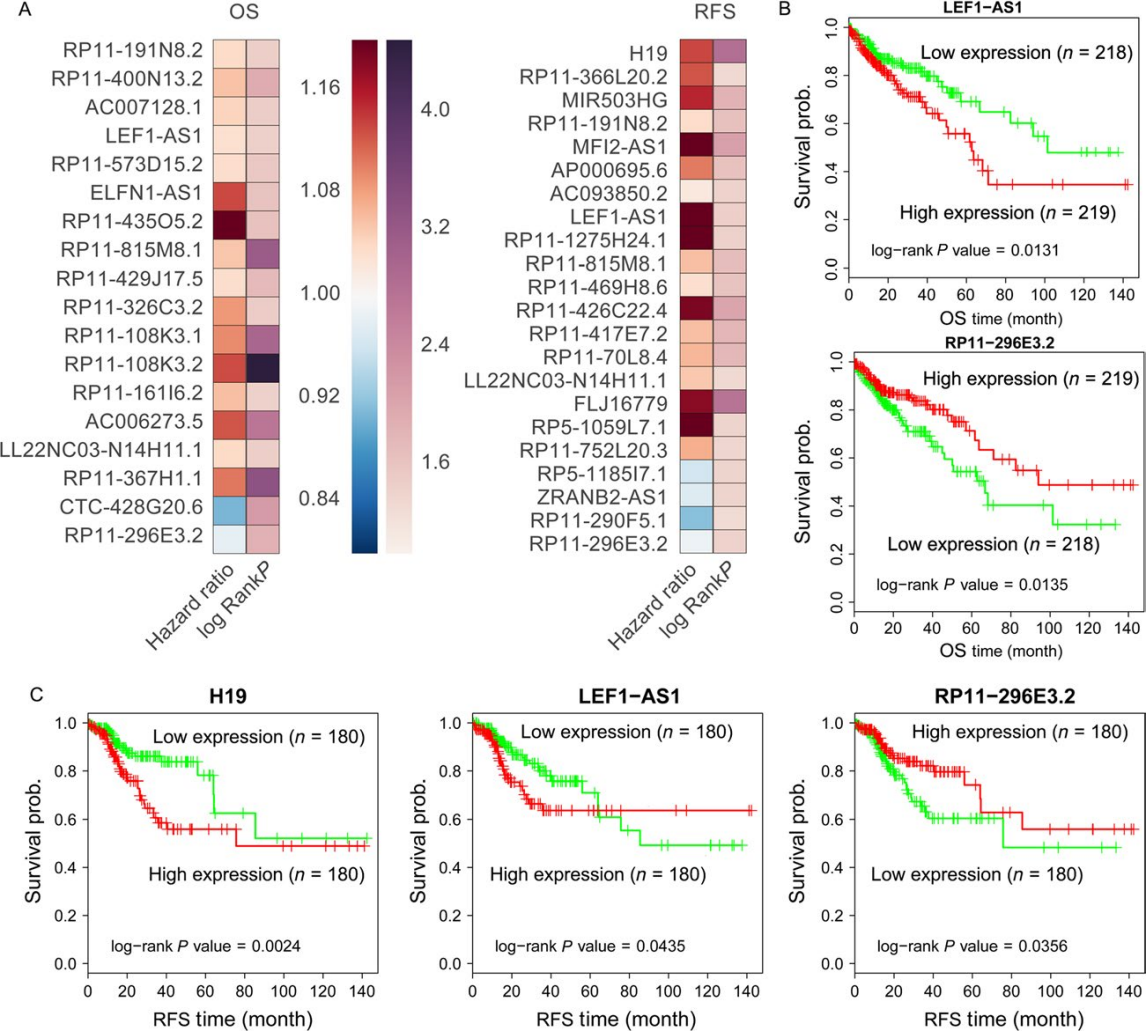
CRC patients OS and RFS associated lncRNAs. (A) Heatmap of the OS and RFS associated lncRNAs log‐rank *P* value and hazard ratio value in CRC. (B) The Kaplan–Meier curve for OS of CRC patients in high‐ (higher H19, LEF1‐AS1 expression) and low‐risk (lower RP11‐296E3.2 expression) groups in the TCGA set, the significant differences were examined using the two‐sided log‐rank test. (C) The Kaplan–Meier curve for RFS of CRC patients in high‐ (higher LEF1‐AS1 expression) and low‐risk (lower RP11‐296E3.2 expression) groups in the TCGA set.

### Identification of survival‐associated lncRNAs in CRC

More and more studies have shown that many lncRNAs expression levels are associated with cancer patients prognosis, and these lncRNAs may be valuable predictors for cancer patients survival time. Therefore, we performed univariable Cox regression analyses to determine whether some of those differently expressed lncRNAs in CRC are related with patients survival. Interestingly, the analysis results showed that 16 upregulated lncRNAs and 2 downregulated lncRNAs are significantly associated with CRC patients poorer OS (log‐rank *P* < 0.05), while 18 upregulated lncRNAs and 3 lncRNAs downregulation are significantly associated with CRC patients RFS (Fig. 3A, Table S4). Take H19, LEF1‐AS1, and RP11‐296E3.2, for example, CRC patients with higher H19 and LEF1‐AS1 expression levels had shorter OS and RFS time, while CRC patients with lower RP11‐296E3.2 expression levels had shorter OS and RFS time (Fig. 3B and C). These results indicate that these CRC survival‐associated dysregulated lncRNAs may be useful candidates for CRC patients prognostic prediction.

### Identification of liver metastasis‐associated lncRNAs in CRC

**Figure 4:**
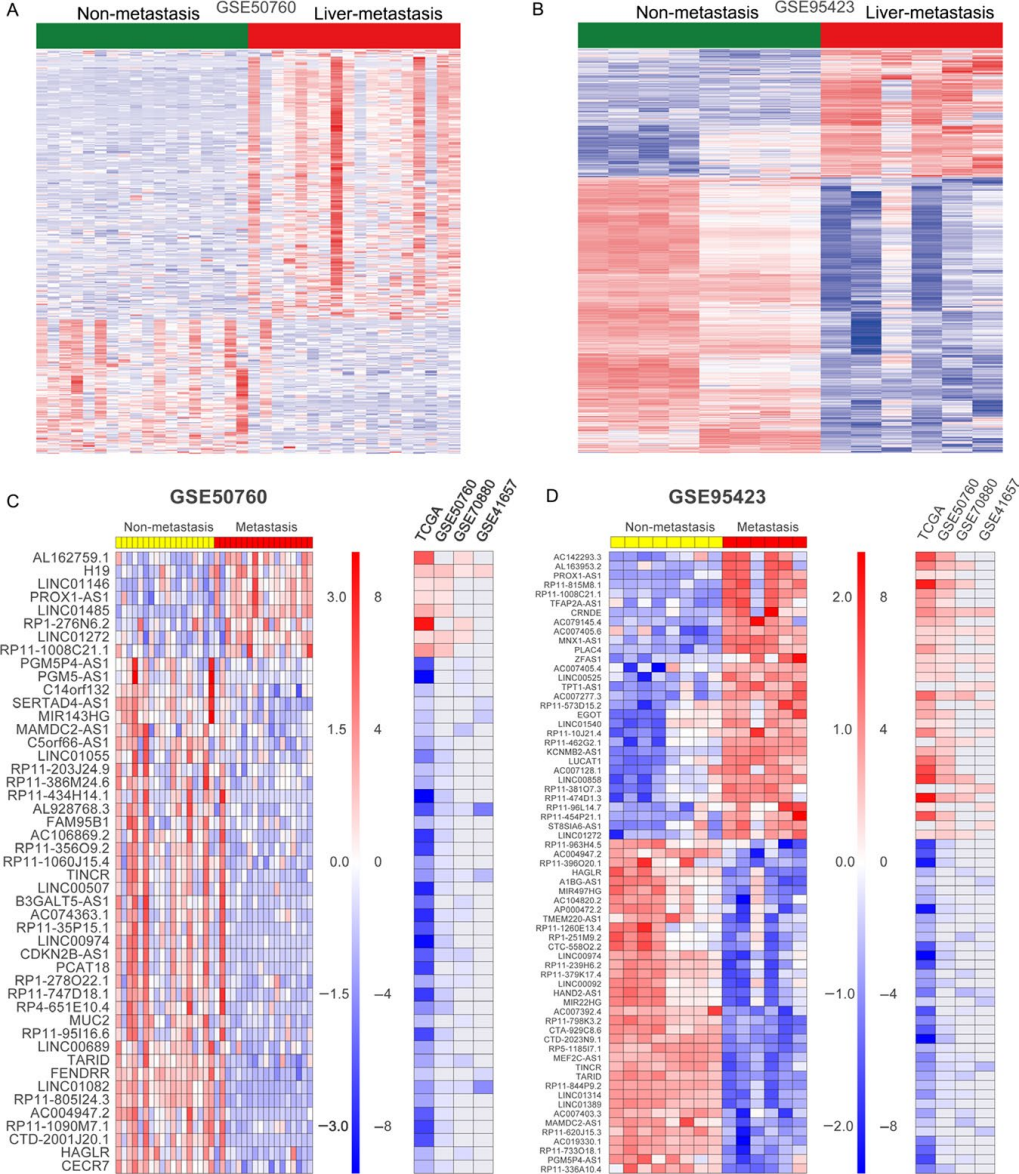
Liver metastasis‐associated lncRNAs in CRC. (A, B) Heatmap of the differentially expressed lncRNAs profiling in CRC tissue samples with liver metastasis compared with primary CRC tissues without liver metastasis were analyzed using the GSE50760 and GSE95423 datasets. (C, D) Heatmap of the significantly upregulated and downregulated lncRNAs in CRC tissue samples with liver metastasis and their fold changes in CRC tissues compared normal tissues in TCGA, GSE50760, GSE41657, and GSE70880 datasets.

It has been well‐known that the CRC patients survival is closely related with the occurrence of metastasis, and liver metastases are observed in nearly 80% of all metastatic CRC. Therefore, exploring new biomarkers of metastatic progression in CRC may lead to earlier diagnosis of patients with liver metastases and improve the CRC patients survival. To identify liver metastasis‐associated lncRNAs in CRC, we download the GSE50760 (18 primary CRC and 18 liver metastasis samples) and GSE95423 (7 primary CRC tissues with liver metastases and 8 CRC tissues without liver metastases) datasets from GEO. Analysis of these data showed that 386 lncRNAs (258 upregulated and 128 downregulated) are dysregulated in GSE50760 dataset and 1681 lncRNAs (532 upregulated and 1149 downregulated) are dysregulated in GSE95423 (Fig. 4A and B, Table S5). Further overlap analysis showed that 36 upregulated lncRNAs in CRC liver metastasis samples are also overexpressed in CRC tissues compared with normal tissues, while 68 downregulated lncRNAs in CRC liver metastasis samples are also decreased in CRC tissues compared with normal tissues (Fig. 4C and D). These findings indicated that lncRNAs are important regulators in CRC cells liver metastasis and may be used as valuable predictors for CRC patients metastasis occurrence.

### Knockdown of LUCAT1 impaired CRC cells invasion

**Figure 5:**
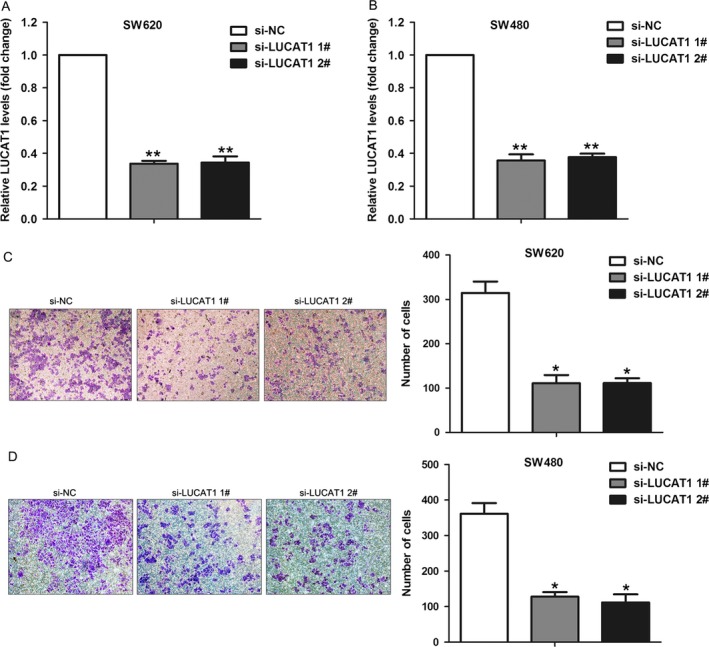
Effect of LUCAT1 knockdown on CRC cells invasion. (A, B) The expression levels of LUCAT1 were detected in SW620 and SW480 cells after transfection with LUCAT1 or negative control siRNAs. (C, D) Transwell assays were used to determine the invasive ability of si‐LUCAT1‐ or si‐NC‐transfected SW620 and SW480 cells. Data represent the mean ± SD from three independent experiments. **P < 0.01, *P < 0.05.

To further determine whether the metastasis‐associated lncRNAs can affect CRC cells invasive ability, upregulated LUCAT1 was chosen for further study. Recent study revealed that LUCAT1 is overexpressed in non–small‐cell‐lung cancer, and promotes cell proliferation by repressing the P57 expression 21. Here, we designed specific siRNAs for LUCAT1, and transfected them into SW620 and SW480 cells to knockdown its expression. The qRT‐PCR results showed that LUCAT1 expression levels were significantly decreased in siRNA‐transfected cells compared with controls (Fig. 5A and B). Next, transwell assays showed that knockdown of LUCAT1 could significantly inhibit SW620 and SW480 cells invasion compared with control cells (Fig. 5C and D). These results indicate that these metastasis‐associated lncRNAs, such as LUCAT1, might play important roles in CRC liver metastasis through promoting cells invasion.

## Discussion

In recent years, increasing research has revealed the critical roles of lncRNAs in cellular process and their dysregulation in human diseases, especially cancer 22, 23. A growing amount of studies have documented that lncRNAs are featured as key regulators in CRC tumorigenesis, metastasis, and survival 24. For example, Yang et al. found that increased GAPLINC expression was positively correlated with larger tumor size, advanced tumor stage, and patients shorter survival, and promoted invasion by targeting SNAI2 through binding with PSF and NONO in CRC 25. Moreover, overexpression of lncRNA PANDAR indicates a poor prognosis for CRC patients and promotes metastasis by regulating EMT pathway 26; higher expression level of H19 is related with a poor prognosis of CRC and promotes tumor cells proliferation by recruiting and binding to eIF4A3 27. Hence, there is an ongoing effort to identify novel aberrantly expressed lncRNAs and determine their possible roles in CRC development and metastasis.

Substantial advances on microarray and next‐generation sequencing technique have revolutionized the domain of cancer research. As a result, RNA‐seq data and microarray‐based expression profiling data provide a more comprehensive and accurate understanding of carcinogenesis and cancer progression at the molecular level. In this study, lncRNA profiling by RNA‐seq and microarray from TCGA and GEO was used to identify a number of novel lncRNAs related to CRC pathogenesis, survival, and liver metastasis. We found that hundreds of lncRNAs are differently expressed in CRC tissues compared with corresponding noncancerous tissues. Moreover, genomic alteration analysis documented that somatic copy number amplification and deletion are involved in some of these lncRNAs dysregulation in CRC tissues, such as PCAT1, CCAT1, and HAND2‐AS1. In addition, survival analyses determine that several lncRNAs increased or decreased expression is significantly associated with CRC patients shorter OS or RFS, suggesting that these lncRNAs may be valuable prediction factors for CRC patients survival.

Cancer cells invasion and metastasis have been the main cause of CRC‐related death, and liver metastasis accounts for more than 70% of all metastatic CRC 28. In the present study, we identified lots of liver metastasis‐associated lncRNAs in CRC by analyzing lncRNA profiling in CRC with liver metastasis compared with that in primary CRC tissues without metastasis. Further combined analysis with the lncRNAs profiling in CRC and nontumor tissues, we for the first time identify LUCAT1 as an oncogenic lncRNA in CRC, which is also associated with CRC cells liver metastasis. LUCAT1 (lung cancer‐associated transcript 1) is a new identified lncRNA, which locates in human chromosome 5q14.3. LUCAT1 overexpression has been found to be associated with poor prognosis in human non–small‐cell‐lung cancer and promote cell growth by epigenetically repressing p21 and p57 expression 21. In this study, we found that knockdown of LUCAT1 could impair CRC cells invasion, which is consistent with our analyses results. These findings indicate that our analyses results can provide valuable lncRNAs candidates for further investigation of lncRNAs function in CRC.

In summary, our findings document that hundreds of lncRNAs were differently expressed in CRC tissues compared with their parental normal tissues. Among these altered lncRNAs, many are significantly associated with CRC patients survival time, and might play critical roles in CRC development and liver metastasis through regulation of cell invasion ability. Our study highlights the important roles of lncRNAs in CRC and may provide useful lncRNA candidates as diagnostic markers and potential targets for CRC therapy. The present study also has a few limitations, for example, only one lncRNA candidate was validated in this study and its underlying mechanism is not investigated. This will need to be further investigated by other researchers.

## Conflicts of Interest

No potential conflicts of interest were disclosed.

## Supporting information


**Table S1**. lncRNAs profiling in TCGA and GEO datasets.

**Table S2**. Dysregulated lncRNAs in TCGA and three GEO datasets.

**Table S3**.Copy number variation analysis of lncRNAs loci in TCGA data.

**Table S4**. Survival associated lncRNAs.

**Table S5**.CRC metastasis associated lncRNAs.
